# Exploration of folate and its derivatives in grains of wheat with different colors

**DOI:** 10.3389/fgene.2025.1549122

**Published:** 2025-04-02

**Authors:** Peng Tian, Yangna Liu, Yingli Cheng, Bin Yang, Yuzhi Wang, Bangbang Wu

**Affiliations:** ^1^ Shanxi Vocational University of Engineering Science and Technology, Taiyuan, Shanxi, China; ^2^ Institute of Hybrid Wheat, Beijing Academy of Agriculture and Forestry Sciences, Beijing, China; ^3^ Institute of Wheat Research, Shanxi Agricultural University, Linfen, Shanxi, China

**Keywords:** wheat, grain color, folates content, genetic factor, QTL

## Abstract

Folate plays essential role in sustaining cell activity, promoting cell growth, and participating in cell division and proliferation. The demand for colored wheat is increasing day by day due to its high content of anthocyanin, iron, zinc, selenium, and other beneficial elements. To investigate the folate content and its derivatives in colored grains wheat, in this study employed a total of 113 wheat varieties (lines) with varying grain colors. The content of four folate derivatives, tetrahydrofolate (THF), 5-methyltetrahydrofolate (5-CH_3_-THF), 5-formyltetrahydrofolate (5-CHO-THF), and 5,10-methylenetetrahydrofolate (5,10-CH^+^THF), in grains cultivated under three different growing conditions were quantified using high performance liquid chromatography (HPLC). The results revealed that the four folate derivatives were distributed among wheat varieties exhibiting varying grain colors, with a coefficient of variation (CV) ranging from 15.34% to 20.10%. Among them, Lin 4179 emerged as a high-folate variety with a total content of 76.00 μg · 100 g^-1^. The contents of 5-CH_3_-THF and 5-CHO-THF in the four folate derivatives accounted for approximately 70% of the total folate content and exhibited a significant correlation with total folate content. The mean total folate level in purple and blue grains was 61.84 and 60.95 μg · 100 g^-1^, respectively, which was significantly higher than that in white (41.93 μg · 100 g^−1^) and red grains (42.40 μg · 100 g^−1^). The genotypic effect is the main factor affecting total folate content, while environmental factors had less impact. Genome-wide association studies (GWAS) identified four major loci associated with folate content on the chromosomes 1B, 4D and 7A, of which *QFac.4D* and *QFac.7A.1* were stated as novel. The results of this study provide valuable insight into the development and breeding of folate biofortified wheat varieties.

## 1 Introduction

Folate (vitamin B9) is a water-soluble B vitamin characterized by tetrahydrofolate and its derivatives. As an essential micronutrient, folate promotes cell growth, maintains cellular activity, and is crucial for cell division and proliferation. It plays a vital role in the normal growth, metabolism, and development of the body (Edelmann et al., 2012). The various derivatives of folate primarily differed in the presence of distinct substituents on the pyrazine ring and the glutamate residue bound to ortho-aminobenzoylglutamate. A deficiency in folate can lead to several health issues, including megaloblastic anemia, atherosclerosis, and colorectal cancer (Zhang and Li, 2019; Field et al., 2018). For pregnant women, inadequate folate intake may result in lower infant birth weight and elevate the risk of congenital conditions such as cleft lip and palate, heart disease, and neural tube malformations (Czeizel et al., 2013). Each year approximately 7.9 million infants are born, with neural tube defects accounting for about 10% of these cases (Rader and Schneeman, 2006; Bhutta and Salam, 2012). Consequently, the necessity for reasonable and effective supplementation of folate to prevent deficiencies has gained attention. While plants and microorganisms can synthesize folate by themselves, whereas animals and humans are unable to synthesize it directly and must rely on dietary intake (Jha et al., 2015). The World Health Organization (WHO) recommended an adult to consume of 400 μg of folate per day, while pregnant women should consume600 μg per day (Rébeillé et al., 2006). Folate deficiency is particularly prevalent in impoverished areas and developing countries, which prompts an increase in research focused on biofortified foods to enhance folate levels in response to the growing concerns regarding folate intake (Jha et al., 2015; Blancquaert et al., 2015; Khalili et al., 2020). Common strategies for addressing folate deficiency include supplementation through pills and processed foods, as well as increasing the consumption of natural folate through a diverse diet (Jha et al., 2015). However, the high costs of synthetic pills and processed foods present significant challenges in underdeveloped regions. Moreover, studies have reported that excessive intake of synthetic folate may increase genome-wide DNA methylation, which in turn increases the risk of colon, rectal, and prostate cancers, along with cognitive impairment (Crider et al., 2012; Rakszegi et al., 2008; Cao et al., 2023). Globally, many people are unable to afford a varied diet beyond staple foods due to economic constraints, dietary habits, or a lack of stable social policies and sustained financial support (Botto et al., 2005). Therefore, implementing breeding or cultivation practices designed to enrich staple food crops with folate represents a health-promoting, widely applicable, and cost-effective approach that could benefit numerous rural and underdeveloped populations. Wheat is one of the most widely cultivated and important food crops worldwide, has been found to contain folate primarily in the forms of tetrahydrofolate (THF), 5-methaltetrahydrofolate (5-CH_3_-THF), 5-formyltetrahydrofolate (5-CHO-THF), and 5,10-methylenetetrafolate (5,10-CH^+^THF) (Riaz et al., 2019; Zheng et al., 2022). Developing wheat varieties that are rich in folate and integrating them into daily diets could significantly reduce the prevalence of folate deficiency (Strobbe and Van Der Straeten, 2017).

According to the difference in the seed coat color, wheat can be classified into common red and white grains, as well as blue and purple colored wheat. Colored wheat is notably rich in essential nutrients, including plant protein, dietary fiber, vitamins, trace elements, and bioactive compounds such as anthocyanins, flavonoids, alkaloids, and phytosterols. These components have been shown to exert beneficial effects on various health conditions, including diabetes, anemia, and cardiovascular diseases (Guo et al., 2013). Colored wheat is often employed in the development of functional health foods, due to its rich nutrient profile. (Zhao et al., 2016). Prior research has highlighted significant variability in folate content among different wheat varieties. Kr et al (1984) reported that the total folate content in wheat from the United States and Canada ranged from 16 to 81 μg · 100 g^−1^. Piironen et al. (2008) found that the folate content in common wheat varied from 32.3 to 77.4 μg · 100 g^−1^, whereas Arcot et al. (2002) recorded a total folate content range of 48–114 μg · 100 g^−1^ for total folate content. In addition, Riaz B. et al. (2019) conducted a study involving 315 wheat varieties in northern China, revealing a total folate content range of 10.15–91.44 μg · 100 g^−1^. Furthermore, Zheng et al. (2022) analyzed the folate content in 262 germplasms from the Chinese wheat mini-core collection, identifying total folate levels ranging from 22.68 to 111.77 μg · 100 g^−1^. Collectively, these studies establish a foundational basis for enhancing the nutritional fortification of folate in wheat species. However, it is noteworthy that the most of the genetic materials selected investigated in these studies were from common wheat varieties, with none of the research conducted specifically on the folate content in colored wheat grains, leaving the potential richness of folate content in colored wheat largely unexplored.

Folates are a group of water-soluble B vitamins (B9), derived from tetrahydrofolate (THF), the most reduced form of folate (Dieter et al., 2014). Folate derivatives consist of three molecular components: the pteridine ring, para-aminobenzoic acid and L-glutamate residues, differing by one-carbon units linked to the N5 and/or N10 positions of tetrahydrofolate (THF) molecules (Gorelova et al., 2017). The pathways for folate and one-carbon synthesis in different species are highly conserved. In plants, the pteridine and para-aminobenzoic acid are synthesized in the cytoplasm and chloroplast, respectively, and then these two precursors are transported to the mitochondria for formation of folates (DellaPenna, 2007). Based on the pathways of folate metabolism in plants, different strategies have been designed to enhance the amount of folate content in crops, including the enhancing of folate and one-carbon metabolism, as well as increasing the folate stability through genetic modification (Jing et al., 2017). The genes and enzymes involved in folate biosynthesis have been identified in several plant species. Prior research has shown that in transgenic Arabidopsis (Hossain et al., 2004) and tomatoes (Díaz et al., 2004), the overexpression of GTP cyclohydrolase I (GCHI), the first committed enzyme in the pteridine biosynthesis, resulted in a 100 to 1, 000 fold increase in pteridine levels and a 2 to 4 fold enhancement of folate content. Rocío I Díaz et al. (2007) subsequently increased folate production in tomato fruit by up to 25-fold by overexpression of aminodeoxychorismate synthase, which catalyzes the first step of para-amino benzoic acid synthesis. Although folate biofortification presents clear potential benefits in fruits commonly consumed in developed countries, the greater prevalence and severe consequences of folate deficiency in developing countries, suggest that the benefits to be derived from folate biofortification in staple crops may have the significantly greater potential (DellaPenna, 2007). It is regrettable that the available quantitative trait loci (QTLs) and candidate genes for folate biofortification in staple crops are quite limited. To date, several QTLs and genes associated with folates in rice (Dong et al., 2013) and maize (Guo et al., 2019) have been identified using linkage-map-based QTL mapping and genome wide association studies (GWAS). However, to the best of our knowledge there is almost no available information regarding the QTLs and genes related to folate in wheat. Thus, marker-assisted breeding and transgenic engineering efforts to enhance the folate levels in wheat are hindered due to the lack of suitable donors. In the present study, HPLC was used to quantify the content of folate and its derivatives in wheat grains. This research aims to analyze the distribution patterns and characteristics of folate across wheat with varying grain colors, while also employing GWAS to identify genetic loci associated with folate content. The findings of this study are expected to provide valuable insights for improving the biological nutritional quality of wheat and the functionalization of main cereal crops.

## 2 Materials and methods

### 2.1 Experimental materials

#### 2.1.1 Plant materials

The plant materials comprised of 113 wheat (*Triticum aestivum* L.) varieties (lines), exhibiting seed coat colors of red, white, blue and purple. The plant materials were collected by our research group. All varieties (lines) were planted at the experimental base of the Wheat Research Institute of Shanxi Agricultural University (36°2′N, 111°18′E, Linfen City) in the years of 2018 (E1), 2019 (E2), and 2021 (E3). Sowing occurred in early October, and harvesting took place in mid-June of the following year. Each variety was planted in four rows, with each 2 m long, and 40 seeds per row. Field management followed standard agricultural practices, and wheat grains were harvested and dried for later use.

#### 2.1.2 Test reagents

Standards for THF, 5-CH_3_-THF, 5-CHO-THF and 5,10-CH^+^THF were procured from Schircks Laboratories, Switzerland, with purities of 98.0%, 98.4%, 99.7%, and 95.1%, respectively. Methanol, acetonitrile and formic acid (chromatographic grade) were purchased from Shanghai MacLean Biochemical Co., Ltd. Biological reagents including β-mercaptoethanol, ascorbic acid, α-amylase and mouse serum were purchased from Aladdin Reagents (Shanghai) Co., Ltd. Potassium dihydrogen phosphate and potassium dihydrogen phosphate trihydrate were obtained in analytically pure form from Shanghai Shenggong Biotechnology Co., Ltd.

#### 2.1.3 Main test instruments

The following instruments were utilized in the experiments: 1,260 Infinity high performance liquid chromatograph (Agilent Technology), JFS-13A laboratory cyclone mill powder (Hangzhou Dacheng Optoelectronic Instrument Co., Ltd.), SB-5200DT ultrasonic degasser (Ningbo Xinzhi Biotechnology Co., Ltd.), Allegra X-30R high-speed low-temperature centrifuge (Beckman Coulter), and MX-S vortex analyzer (SCILOGEX).

### 2.2 Preparation of folate standard stock solution

Accurately weigh 5.0 mg each standard, including THF, 5-CH_3_-THF, 5-CHO-THF and 5, 10-CH^+^THF standards, and transfer these samples into 10 mL brown volumetric flasks. Subsequently, dilute to the mark with a volume of 20 mmol L^−1^ phosphate buffer, which contains a 1% mass volume fraction of ascorbic acid solution and a 0.1% (v/v) β-mercaptoethanol, adjusted to a pH of 7.0. This preparation results in a folate standard stock solution with a mass concentration of 0.5 mg mL^−1^.The standard solution of the standard reserves of folate is gradually diluted to 2.5, 5.0, 10.0, 25.0, 50.0, 100.0 ng mL^−1^ for the production standard curve. The specific dilution concentrations are shown in Figure 2. The resulting standard solution should be stored at −20°C to maintain stability. It is imperative that this procedure be conducted under dark conditions.

### 2.3 Sample preparation and folate determination

The wheat kernels were dried to a moisture content of 14%, and subsequently ground into a fine powder (passed through a 100-mesh sieve) using a cyclone mill. An aliquot of 0.10 g of whole wheat flour was accurately weighed and combined with 1.0 mL of freshly prepared phosphate buffer solution. The resulting mixture was thoroughly mixed using a vortex mixer. To prevent the mutual conversion of folate derivatives, the mixture was first boiled in water for 5 min, after which it was rapidly cooled on ice. Subsequently, 50 μL of α-amylase solution was added and the mixture was well mixed before being incubated at 37°C for 30 min, with shaking every 10 min. Following this incubation period, the α-amylase was inactivated in a boiling water bath for 3 min and then quickly cooled on ice. After the addition of 20 μL of mouse serum, the cells were incubated at 37°C for 2 h, with shaking every 20 min. Upon completion of the incubation with mouse serum, the cells were again subjected to a boiling water bath for 3 min, cooled on ice, and centrifuged at 12,000 r·min^-1 for 10 min at 4°C. The supernatant was subsequently transferred to a 3 kD ultrafiltration centrifuge tube and centrifuged for 30 min at 12,000 rpm ^-1 and 4°C. Finally, the supernatant was stored at −20°C for later use.

### 2.4 Chromatum parameters and methodology verification

The Agilent 1,260 high-performance liquid chromatograph utilized in this study is controlled by the Agilent Chem Station. The system features a UV detector, and the sample loop volume is set at 20 μL. The chromatographic column employed is Akzo Nobel 100–5 C18 analytical column, with the dimensions of 50 mm × 2.1 mm. Mobile phases of A and B consist of water and acetonitrile, respectively, both of which are supplemented with 0.1% by volume of formic acid. Prior to use, the mobile phase was filtered through a 0.22 μm organic filter and degassed using an ultrasonic degasser for a duration of 20 min. The elution gradient was maintained for 10 min, with a flow rate of 0.2 mL min^−1^, while ultraviolet detection was conducted at a wavelength of 280 nm. The injector and column oven temperatures were controlled at 4°C and 25°C, respectively. The initial elution conditions comprised 95% mobile phase A and 5% mobile phase B. Over the subsequent 2 min, the proportion of mobile phase B increased from 5% to 9%. At the time of 8 min, the proportion of mobile phase B was raised from 9% to 9.6%, this was followed by a decrease back to the initial proportions over the next 2 min, ultimately achieving equilibrium.

Folate standards were utilized to determine the optimal elution conditions and retention times. Each derivative of folate present in the samples was identified according to its retention time. The peak areas were automatically integrated using the Agilent Chem Station chromatographic system. Quantification of the samples was conducted using the external standard method with each sample analyzed was in triplicate, and the average value was recorded. After the extraction of folate was extracted from the varieties Yaomai 33 and Jinmai 47, treatments with and without the addition of standard products were utilized as controls. The mean of three measurements was recorded to evaluate the spiked recovery rate of each folate derivative. For the Yaomai 33 flour sample, five repeated extractions were performed, and the content of folate derivatives was recorded. Subsequently, the relative standard deviation (RSD) was calculated to assess the precision and accuracy of the detection method.

### 2.5 Data processing

The broad-sense heritability (*H* ^*2*^) is defined as *H* ^*2*^ = V_G_/(V_G_ + V_E_), where V_G_ and V_E_ represent the genetic and environmental variance, respectively. JMP Pro16 (2022 JMP Statistical Discovery LLC) was used to obtain the best linear unbiased prediction (BLUP) and calculate broad-sense heritability (*H* ^*2*^). SPSS 26.0 (IBM SPSS Statistics; IBM Corp., Armonk, NY, United States) was utilized for correlation analysis and the comparison of significant differences, and Origin 2022b was used to generate high-performance liquid chromatograms and standard curves.

### 2.6 Genome wide association analysis

A 90K single nucleotide polymorphism (SNP) chip was used for genotype identification, and the effective SNPs after filtering were used for subsequent analysis. The mixed linear model (MLM) Q + K implemented in TASSEL5.0 software was used to conduct GWAS for folate content. The threshold p-value was determined by comparing the -log_10_ (p-value) derived from the QQ plot (Quantile-Quantile plot) with the expected values. Using R packages (qqman, tidyverse, data.table) to visualize the dataset results and generate a manhattan plot. The linkage disequilibrium (LD) among the SNP markers was observed to extend across each chromosome. The software PLINK 1.9 was used to calculate the linkage disequilibrium value *r* ^2^ between SNP loci on each chromosome, with the extended region defined as the LD quantitative trait loci (QTL) interval based on *r* ^2^ = 0.20. The physical locations of the SNP markers were obtained from the Chinese Spring reference genome IWGSC v1.0.

## 3 Results and analysis

### 3.1 Method and verification of results

**FIGURE 1:**
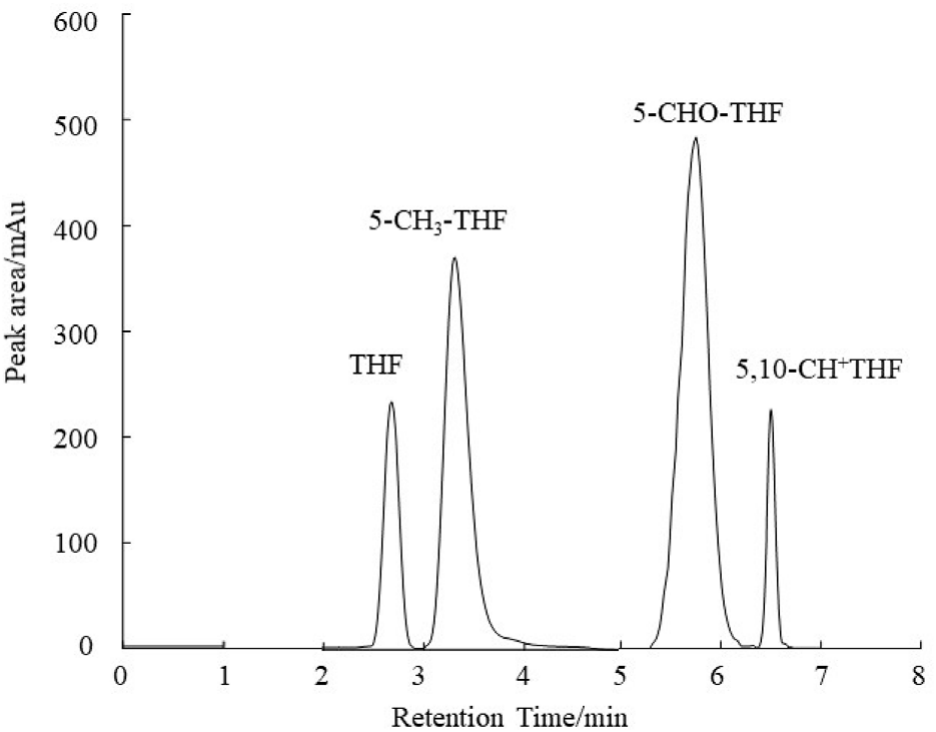
HPLC chromatograms of the folate standard mixture.

**FIGURE 2:**
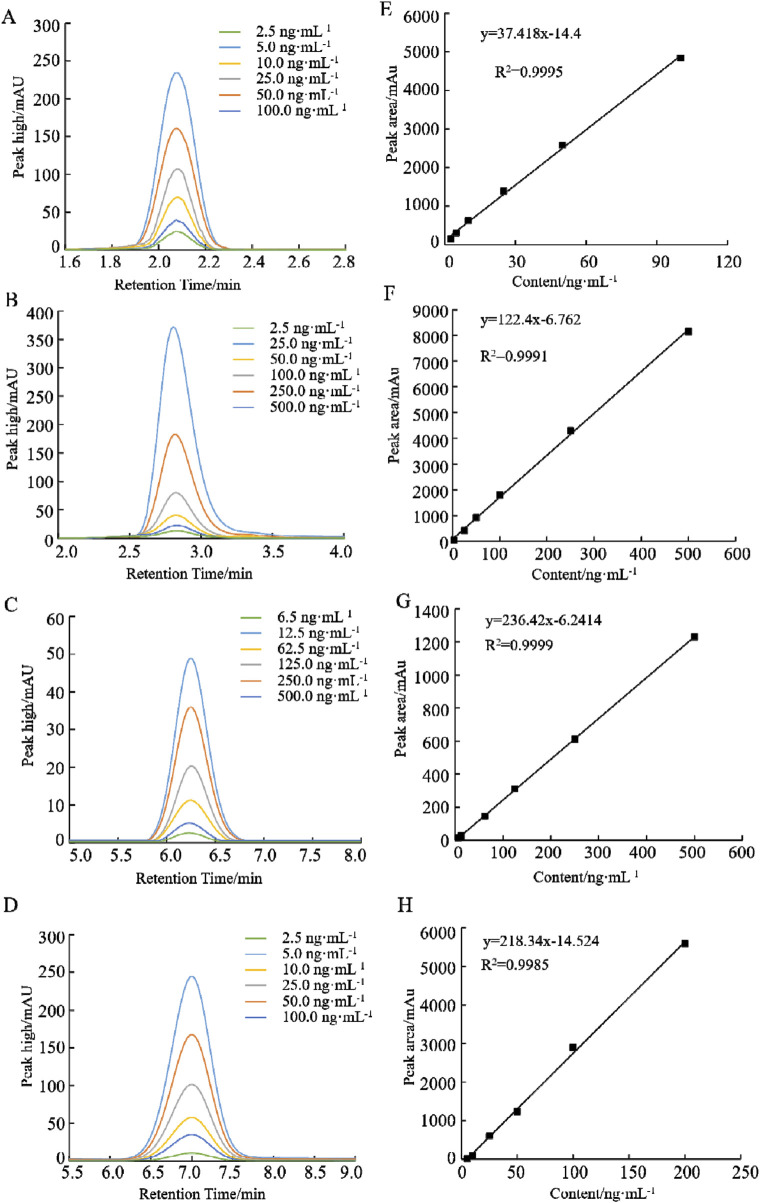
HPLC chromatograms of folates with different concentrations and standard curves of peak areas and folate contents. **(A, E)** THF, **(B, F)** 5-CH_3_-THF, **(C, G)** 5-CHO-THF, and **(D, H)** 5,10-CH^+^THF.

The standard substances THF, 5-CH_3_-THF, 5-CHO-THF and 5,10-CH^+^THF were dissolved in a phosphate buffer solution (20 mmol L^−1^, pH 7.0). The retention times for THF, 5-CH_3_-THF, 5-CHO-THF and 5,10-CH^+^THF were determined to be 2.88 min, 3.28 min, 5.74 min and 6.53 min, respectively. As illustrated in Figure 1, the chromatograms for the different folate derivatives exhibited stable baselines, with the standard substances demonstrating well-defined peak shapes, and an absence of significant tailing. The concentrations of the different folate derivative standard solutions were injected in order from low to high, and quantitative analysis was performed using the peak area external standard method. The regression equation of the standard curve exhibited favorable linearity, facilitating the determination of folate derivative content in the sample by substituting the corresponding retention times and peak areas into the respective standard curve as shown in Figure 2.

The concentrations of various folate derivatives were quantified in Yaomai 33 and Jinmai 47, both with and without the addition of spiked samples. The results indicated that the recoveries of the spiked folate derivatives consistently above exceeded 95%, thereby further demonstrating the accuracy and reliability of the detection method used (Table 1).

**TABLE 1:** The spiked recoveries of four folate derivatives.

Material	Derivatives	Sample content/(μg · 100 g^−1^)	Added/(μg · 100 g^−1^)	Detection/(μg · 100 g^−1^)	Recovery rate/%
Yaomai 33	THF	3.84	6	9.41	95.63
	5-CH_3_-THF	10.14	20	29.54	98.01
	5-CHO-THF	13.98	20	33.59	98.85
	5, 10-CH^+^THF	4.54	8	12.15	96.89
Jinmai 47	THF	3.95	6	9.48	95.28
	5-CH_3_-THF	10.23	20	29.12	97.33
	5-CHO-THF	16.11	20	34.55	95.68
	5, 10-CH^+^THF	5.32	8	12.88	96.70

Yaomai 33 was selected for the repeated determination of the content of each folate derivative in order to verify the precision of the method. The results of the determinations are presented in Table 2. The findings indicated that the RSD of the folate derivative content was less than 4%, suggesting that the precision and repeatability of the extraction method were satisfactory.

**TABLE 2:** Relative standard deviations of four folate derivatives (n = 5).

Material	Derivatives	Sample content/(μg·100 g^-1^)					Average/(μg·100 g^-1^)	RSD/%
		1	2	3	4	5		
Yaomai 33	THF	3.87	3.59	3.78	3.91	3.59	3.75	3.62
	5-CH_3_-THF	10.37	10.62	9.97	10.29	10.74	10.40	2.59
	5-CHO-THF	13.87	15.45	14.26	14.47	14.88	14.59	3.71
	5, 10-CH^+^THF	4.68	4.46	4.23	4.34	4.22	4.39	3.89

### 3.2 Analysis of folate content in wheat grains

Folate was extracted from materials harvested under three distinct environments and quantified. Four folate derivatives were identified in wheat with varying grain colors. The total folate content across different wheat varieties varied widely, with a coefficient of variation (CV) ranging from 15.34% to 20.10%. The folate content recorded across the three environments ranged from 34.49–76.02 μg · 100 g^−1^, 32.29–74.50 μg · 100 g^−1^, and 33.60–76.67 μg · 100 g^−1^, respectively, with average values of 61.38 μg · 100 g^−1^, 57.84 μg · 100 g^−1^, and 59.41 μg · 100 g^−1^, respectively. The three varieties exhibiting the highest total folate content in the E1 environment were Taixue 24, Lin 4179, and Kemai 671; in the E2 environment, they were Shimai 211, Taixue 24, and Lin 4179; and in the E3 environment, they were Lin 4179, Shimai 211, and Bingnongzi 1. Notably, the total folate content of Lin 4179 in all three environments exceeded 70 μg · 100 g^−1^, categorizing it as a variety with a high folate content. Furthermore, the 5-CH_3_-THF content of Lin 6,080 was the highest among all evaluated varieties (Supplementary Table 1).

### 3.3 Effect of grain color on folate content in wheat

According to the seed coat color, the materials were divided into red, white, blue and purple grains. A comparative analysis was conducted on the folate and its derivatives present in wheat grains of varying colors, with particular emphasis on the influence of grain color on folate levels. The results showed that the total folate content in purple-grain and blue-grain wheat was 61.84 and 60.95 μg · 100 g^−1^, respectively, whereas the total folate content in the white-grain and red-grain wheat were 41.93 and 42.40 μg · 100 g^−1^, respectively. Notably, the total folate contents in purple-grain and blue-grain wheat were significantly higher than those in white-grain and red-grain wheat. Additionally, the average contents of 5-CH_3_-THF and 5-CHO-THF in purple-grain and blue-grain wheat were significantly higher than those in white-grain and red-grain wheat, while the contents of THF and 5-CHO^+^THF did not exhibit significant difference. However, no significant differences were observed in the contents of THF, 5-CH_3_-THF, 5-CHO-THF, 5,10-CH^+^THF and total folate between white-grain and red-grain wheat, nor between purple-grain and blue-grain wheat (Table 3). In conclusion, the concentrations of 5-CH_3_-THF, 5-CHO-THF and total folate were significantly higher in purple and blue varieties than those in white and red varieties.

**TABLE 3:** Variation of folate contents among grain coat color genotypes.

Type	THF (μg·100 g^−1^)	5-CH_3_-THF (μg·100 g^−1^)	5-CHO-THF (μg·100 g^−1^)	5, 10-CH^+^THF (μg·100 g^−1^)	Total folate (μg·100 g^−1^)
White-grain	4.39 a	16.68 a	16.29 a	4.56 a	41.93 a
Red-grain	4.11 a	17.11 a	16.39 a	4.79 a	42.40 a
Blue-grain	3.93 a	23.44 b	27.61 b	5.47 a	60.95 b
Purple-grain	4.15 a	25.03 b	27.04 b	5.38 a	61.84 b

Different lowercase letters indicate that there are significant differences (*P* < 0.05).

### 3.4 Correlation of folate contents and seed coat colors

The correlation analysis conducted between different folate derivatives and the total folate content revealed a significant association between 5-CH_3_-THF and 5-CHO-THF. Additionally, a significant correlation was observed between 5-CH_3_-THF and 5-CHO-THF. Furthermore, there was a significant correlation between seed coat color with 5-CH_3_-THF, 5-CHO-THF and total folate content, while there was no significant correlation between THF and 5, 10-CH^+^THF with respect to both total folate content and seed coat color (Table 4).

**TABLE 4:** Correlation between different folate derivatives, grain coat color and total folate content.

Folate derivatives	THF	5-CH_3_-THF	5-CHO-THF	5, 10-CH^+^THF	Total folate
5-CH_3_-THF	−0.083				
5-CHO-THF	−0.191	0.634^**^			
5, 10-CH^+^THF	−0.127	0.0073	0.130		
Total folate	−0.017	0.878^**^	0.908^**^	0.130	
Seed coat color	0.052	0.642^**^	0.557^**^	0.106	0.645^**^

* and ** indicate significant correlation at 0.05 and 0.01 levels, respectively.

### 3.5 Effects of genotype, environment and their interaction on folate content

The effects of genotype, environment and their interaction on folate content were systematically investigated by quantifying the contents of various folate derivatives as well as the total folate content in grains cultivated under three distinct environments. The findings indicated that both the genotype effect (G) and the genotype-environment interaction effect (G × E) on the total folate content in grains were highly significant (Table 5). Genotype exerted significant effects on the levels of four folate derivatives, whereas environmental conditions demonstrated significant effects solely on 5-CH_3_-THF and 5,10-CH^+^THF. The order of influence on THF, 5-CHO-THF and total folate content was observed as G > G × E > E, while the order of influence on changes in 5-CH_3_-THF was G > E > G × E, and the order of influence on 5,10-CH^+^THF was G × E > G > E. These results suggest that genotype serves as the main factor affecting folate content, while environmental factors having little effect.

**TABLE 5:** Effect of genotype, environment, and their interaction on folate content.

Folate derivatives	Genotype (G)	Environment (E)	G × E	(*h* ^*2*^)
THF	57.973**	9.08	32.947**	0.78
5-CH_3_-THF	38.291**	31.147**	30.562**	0.89
5-CHO-THF	92.404**	0.087	7.509**	0.96
5, 10-CH^+^THF	24.766**	19.467**	55.767**	0.70
Total folate	84.159**	7.206	8.635**	0.94

** represents the significance level calculated at *P* ≤ 0.01.

### 3.6 GWAS analysis of folate content

**FIGURE 3:**
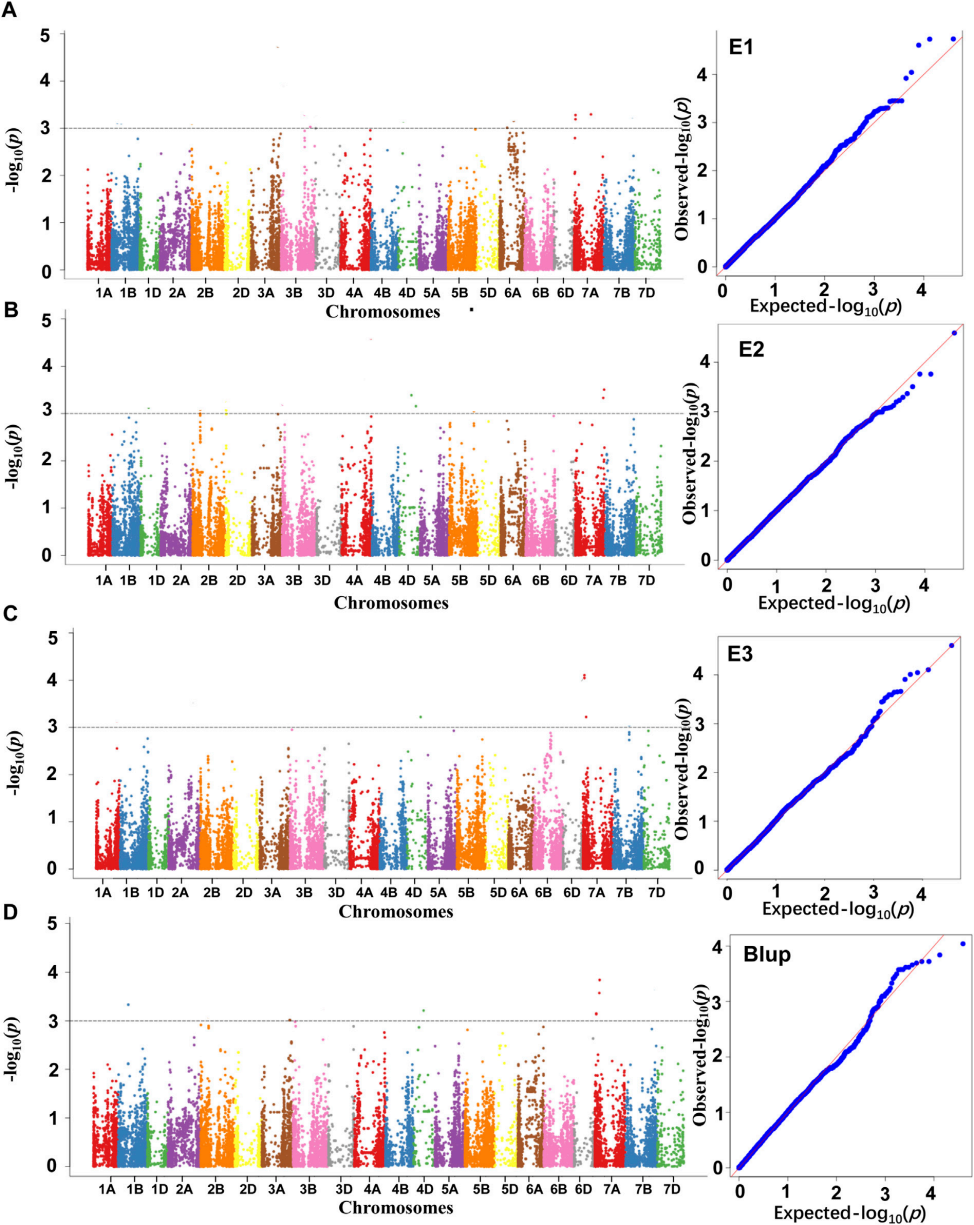
The GWAS result of the folate content from different environments **(A**-E1, **B**-E2, **C**-E3, **D**-Blup**)**. The black dashed line represents the threshold for the significance of marker trait association.

A GWAS of folate content was conducted using the MLM model, resulting in the identification of nine significantly associated SNP markers, referred to as marker-trait associations (MTAs) distributed on chromosomes 1B, 4D, and 7A. Specifically, three MTAs were identified on chromosome 7A in E1 (Figure 3A), three MTAs were found in E2, located on chromosomes 4D and 7A, respectively (Figure 3B). Four MTA was detected in E3, which is located on chromosome 4D and 7A (Figure 3C). Under BLUP, six MTAs were identified on chromosomes 1B, 4D, and 7A (Figure 3D).

Based on the size of the LD interval for each chromosome, the nine MTAs correspond to four QTL loci, designated as *QFac.1B, QFac.4D, QFac.7A.1* and *QFac.7A.2,* in which *QFac.7A.1* and *QFac.7A.2* were detected across three environments (Table 6).

**TABLE 6:** Summary of associated loci with traits by genome-wide association study (GWAS).

Loci	Chr.	Interval (MB)	Environment	Peak SNP	*P*-value	*R* ^2^ (%)
*QFac.1B*	1B	135.25-158.25	BLUP	*1B_146746777*	6.58E^-05^	5.69
*QFac.4D*	4D	648.92-660.63	BLUP	*4D_653919298*	5.67E^-05^	8.43
			E2	*4D _655632858*	5.60E^-05^	7.81
*QFac.7A.1*	7A	75.63-95.65	E1, E2, BLUP	*7A_85627950*	6.31E^-05^	6.24
			E1, E3, BLUP	*7A_85658392*	4.86E^-05^	10.45
*QFac.7A.2*	7A	729.71-765.19	E2	*7A_739708412*	4.09E^-05^	11.88
			E1	*7A_741083088*	4.43E^-05^	10.51
			BLUP	*7A_741778749*	4.95E^-05^	10.33
			BLUP	*7A_755187469*	4.33E^-05^	10.11

**FIGURE 4:**
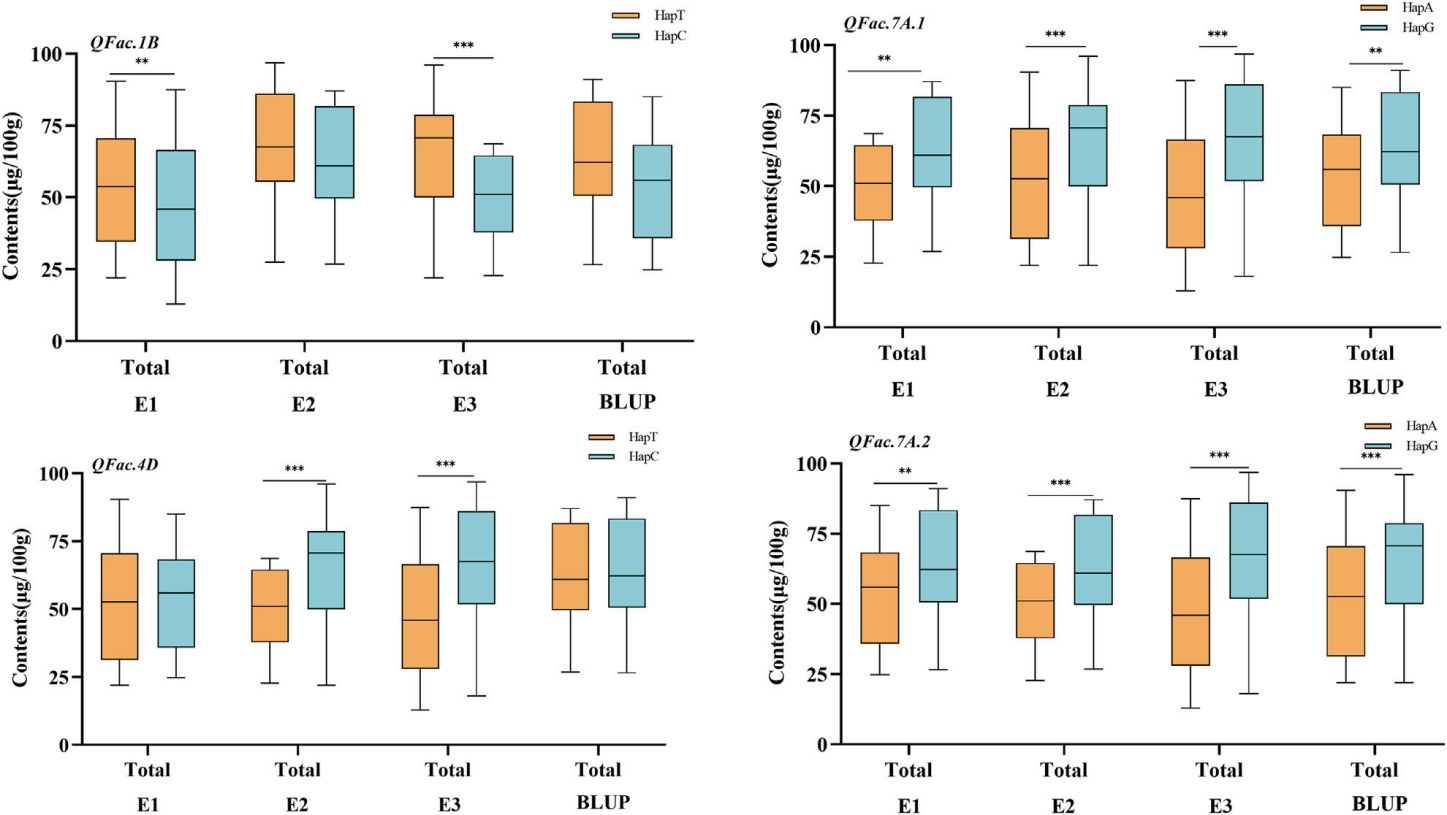
Effect of QTL associated with folate The asterisks ** and *** represent significance levels at p < 0.01 and p < 0.001.

There were significant differences among the different allelic variations of the four loci related to total folate content. *QFac.7A.2* (A/G)·effect was the largest, and the HapG content was 23.41% higher than HapA, followed by *QFac.7A.1* (A/G), and the HapG content was 20.16% higher than HapA (Figure 4).

## 4 Discussion

The current methodologies for the determination of folic acid primarily encompass microbiological techniques, UV-visible spectrophotometry, fluorescence analysis, and high-performance liquid chromatography (Guo et al., 2003; Pandrangi and LaBorde, 2004; Chang et al., 2010; Su, 2011; Wu et al., 2022). The microbial method, a classical approach for assessing folate contents, quantifies folate based on its absorption by microorganisms within the sample matrix, thereby effectively measuring the total folate content. Although this method is relatively accurate and cost-effective, it is limited by its inability to differentiate among various folate derivatives. Furthermore, the method is characterized by an extended experimental cycle and limited repeatability of results across different batches. The UV-spectrophotometric method is favored due to its operational simplicity and minimal extraction requirements, but it is limited to quantifying the total amount of folate and fails to differentiate between various components. In addition, the type of solvent can easily cause large errors. Although fluorescence analysis exhibits greater sensitivity compared to ultraviolet spectrophotometry, the intrinsic fluorescence intensity of folate is relatively low. As a result, sensitizers such as potassium permanganate, fenton’s reagent, or potassium persulfate are frequently used, which may often lead to increase measurement errors. HPLC is widely accepted for its effective separation capabilities and high sensitivity towards different folate derivatives, and it can be integrated with several analytical technologies. Currently, HPLC is the most widely used method for folate detection and has been successfully applied to quantify the content of different folate derivatives in fruits, vegetables and crops (Gujska and Kuncewicz, 2005; Zhang et al., 2005; Póo-Prieto et al., 2006). Therefore, this study used HPLC to determine the folate content in wheat grains. Prior research has demonstrated that four folate derivativesviz THF, 5-CH_3_-THF, 5-CHO-THF and 5, 10-CH^+^THF, account for more than 90% of the total folate content in wheat grains, representing the main forms of folate present in this matrix (Riaz et al., 2019; Zheng et al., 2022; Pfeiffer et al., 1997; Upadhyaya et al., 2017). Variability in germplasm sources and analytical methodologies has led to slight differences in the previously reported folate content in wheat grains. Piironen et al. (2008) used a microbial method to quantify the total folate content of 150 wheat varieties grown at the same environment, revealing a range from 32.3–77.4 μg · 100 g^−1^. Additionally, Piironen et al. (2008) employed HPLC to analyze the content of different folate derivatives in nine wheat varieties, identifying 5-CHO-THF as the main form of folate in wheat. Riaz B et al. (2019) also applied HPLC to assess the folate content in 360 wheat varieties, ranging from 10.15 ± 2.86 to 91.44 ± 5.64 μg · 100 g^−1^. The main forms of folate identified in wheat grains were found to be 5-CH_3_-THF and 5-CHO-THF, which is consistent with the findings of this study. However, the research conducted by Riaz B. et al. (2019) indicated that the number of common varieties across different environments was relatively limited. In this research study, Lin 4179 was selected due to its total folate content exceeding 65 μg · 100 g^-1^ across multiple environments, while Lin 6,080 exhibited significantly higher levels of 5-CH_3_-THF compared to other varieties. These materials provide valuable germplasm resources for the breeding of biofortified wheat varieties.

Studies have indicated that darker colored wheat grains contain significantly higher levels of anthocyanins, with concentrations that can be 2 to 6 times greater than those found in conventional wheat. Additionally, the quality traits of blue-grain and purple-grain wheat varieties are generally superior to those of white-grain varieties (Li et al., 2003). However, there is a lack of research investigating the relationship between folate content and seed coat color. By comparing the folate content of 113 wheat grains exhibiting varying seed coat colors, this study revealed that the total folate content in blue grain and purple grain wheat was significantly higher than that in red grain and white grain varieties. However, no significant difference was observed in total folate content between the red grain and white grain varieties, nor between blue grain and purple grain wheat varieties. This discrepancy may be attributed to the presence of a greater concentration of secondary metabolites in the seed coat and endosperm of blue and purple grain wheat, which may facilitate to the accumulation of various folate derivatives. It is important to consider that the variation in folate content among different grain colors may also be influenced by limited number of varieties included in this study. Shewry et al. (2010) planted the same wheat variety across different countries and measured the folate content in the grains after harvest. They found that the environmental factors exerted significant influence on the total folate content, whereas the genotype had comparatively lesser impact. Similarly, Riaz et al. (2019) found that in addition to being affected by the environment, the folate content in wheat was also significantly affected by genes and gene × environment interactions. This finding is inconsistent with the conclusion of the current research, which shown that genotype is the key factor influencing folate content. This discrepancy may be because of Shewry and Bisma’s research used a limited number of materials or common varieties across different environments, which may not have accurately analyzed the effects of genotype and environment on wheat folate content. In contrast, Zheng et al. (2022) used Chinese wheat mini-core collection as material to measure the folate content of grains harvested under varying environmental conditions and found that genotype was the main factor affecting folate content. The present research measured the folate content of 113 wheat grains with different grain colors after harvest across various environments and reached a consistent conclusion.

Folate biofortification of staple crops through metabolic engineering provides a good alternative in the battle against folate deficiency, especially for the remote and poor populations (Shohag et al., 2020). Since most staple crops are poor sources of folates, the biofortification of staple crops with folate can be achieved by two approaches: through conventional breeding or through genetic engineering. Crops breeding can be very time-consuming, hence it need be facilitated and accelerated by quantitative trait loci mapping, in combination with marker-assisted breeding (Blancquaert et al., 2014). To date, the genetic loci and candidate genes concerning folate biosynthesis in staple crops are considerably limited, rarely can be utilized in crops breeding. With the rapid development of sequencing technology and the successful research and development of SNP arrays, genome-wide association study (GWAS) has become one of the main means to mine quantitative trait gene loci (Alqudah et al., 2020). The present study is the first step towards folate-candidate regions identification in bread wheat, four major loci associated with folate content on the chromosomes 1B, 4D and 7A were found by GWAS, of which QFac.4D and QFac.7A.1 were stated as novel. The development of corresponding molecular makers will be an efficient way to promote efforts for breeding higher-folate-level varieties of wheat. These findings provide valuable insights for future breeding information and promotion of wheat varieties that are biofortified with folate.

## Data Availability

The original contributions presented in the study are included in the article/Supplementary Material, further inquiries can be directed to the corresponding authors.
